# Exploration of low-frequency allelic variants of SARS-CoV-2 genomes reveals coinfections in Mexico occurred during periods of VOCs turnover

**DOI:** 10.1099/mgen.0.001220

**Published:** 2024-03-21

**Authors:** Rodrigo García-López, Blanca Taboada, Selene Zárate, José Esteban Muñoz-Medina, Angel Gustavo Salas-Lais, Alfredo Herrera-Estrella, Celia Boukadida, Joel Armando Vazquez-Perez, Bruno Gómez-Gil, Alejandro Sanchez-Flores, Carlos F. Arias

**Affiliations:** 1 Departamento de Genética del Desarrollo y Fisiología Molecular, Instituto de Biotecnología, Universidad Nacional Autónoma de México, Cuernavaca, Morelos, Mexico; 2 Posgrado en Ciencias Genómicas, Universidad Autónoma de la Ciudad de México, Mexico City, Mexico; 3 Coordinación de Calidad de Insumos y Laboratorios Especializados, Instituto Mexicano del Seguro Social, Mexico City, Mexico; 4 Laboratorio Nacional de Genómica para la Biodiversidad-Unidad de Genómica Avanzada, Centro de Investigación y de Estudios Avanzados del IPN, Irapuato, Guanajuato, Mexico; 5 Centro de Investigación en Enfermedades Infecciosas, Instituto Nacional de Enfermedades Respiratorias Ismael Cosío Villegas, Mexico City, Mexico; 6 Laboratorio de Biología Molecular de Enfermedades Emergentes y EPOC, Instituto Nacional de Enfermedades Respiratorias Ismael Cosío Villegas, Mexico City, Mexico; 7 Centro de Investigación en Alimentación y Desarrollo AC, Unidad Mazatlán, Mazatlán, Sinaloa, Mexico; 8 Unidad Universitaria de Secuenciación Masiva y Bioinformática, Instituto de Biotecnología, Universidad Nacional Autónoma de México, Cuernavaca, Morelos, Mexico

**Keywords:** SARS-CoV-2, coinfections, genomic surveillance, Mexico, viral quasispecies

## Abstract

A total of 14 973 alleles in 29 661 sequenced samples collected between March 2021 and January 2023 by the Mexican Consortium for Genomic Surveillance (CoViGen-Mex) and collaborators were used to construct a thorough map of mutations of the Mexican SARS-CoV-2 genomic landscape containing Intra-Patient Minor Allelic Variants (IPMAVs), which are low-frequency alleles not ordinarily present in a genomic consensus sequence. This additional information proved critical in identifying putative coinfecting variants included alongside the most common variants, B.1.1.222, B.1.1.519, and variants of concern (VOCs) Alpha, Gamma, Delta, and Omicron. A total of 379 coinfection events were recorded in the dataset (a rate of 1.28 %), resulting in the first such catalogue in Mexico. The most common putative coinfections occurred during the spread of Delta or after the introduction of Omicron BA.2 and its descendants. Coinfections occurred constantly during periods of variant turnover when more than one variant shared the same niche and high infection rate was observed, which was dependent on the local variants and time. Coinfections might occur at a higher frequency than customarily reported, but they are often ignored as only the consensus sequence is reported for lineage identification.

## Graphical abstract

Coinfections occurring within a single sample can be detected by exploring all mutations in a genomic set as these have full profiles commonly observed in lower prevalence.

**Impact Statement**

The study of IPMAVs allows for detecting coinfections occurring as mostly complete mutational profiles, as secondary variants that are present in the sample when multiple high-prevalence variants circulate. In Mexico, a succession of turnover events of different variants of concern (VOCs) of SARS-CoV-2 drove the two largest epidemiological surges of COVID-19 in Mexico in 2021. Such high transmission periods, when multiple variants were circulating, fostered coinfections. This knowledge will prove beneficial to studying the emergence of recombinant variants that might have clinical relevance.

## Data Summary

All sequences of samples bearing putative coinfections are available at NCBI’s SRA database under Bioproject PRJNA1008275 and Accession numbers: PRJNA1008275-SRR25735865. The GISAID accession numbers of all genomes produced by CoViGen-Mex and collaborators are provided under EPI_SET ID: EPI_SET_231031gz with doi: 10.55876/gis8.231031gz. Both types of identifiers and their associated metadata can be found in Table S1, available in the online version of this article.

## Introduction

The years 2019–2023 have been marked by the worldwide pandemic of the coronavirus 2019 disease (COVID-19), the respiratory disease caused by the severe acute respiratory syndrome coronavirus 2 (SARS-CoV-2). For this virus, a variant is the most specific level of classification, defined as a viral genome containing a specific set of mutations that allows its association with a phylogenetic lineage, and it is commonly designated using the Pango nomenclature [1].

In Mexico, the first confirmed case of COVID-19 was detected on 27 February 2020 [2]. As of the time of writing, 7 624 493 SARS-CoV-2-positive cases and 333 714 deaths have been registered by Mexican Official authorities [2]. The country has traversed six different epidemiological surges (waves) and has registered more than 87 657 genomes in the EpiCoV data base, including 680 different variants thus far [3].

Throughout more than 3 years of continuous evolution in humans during the emergency phase of the pandemic around the world, over 192 010 different nucleotide substitutions and insertions/deletions (the latter are counted by single position) have been recorded along SARS-CoV-2’s 29.9 kb genome [4]; producing 127 272 amino acid substitutions.

International protocols for the genomic surveillance of SARS-CoV-2 have been standardized for second generation high-throughput sequencing with platforms such as Illumina’s [5]. These produce several hundreds to thousands of short sequencing reads per genomic position, as libraries are constructed from multiple cDNA molecules derived from different virions, which may not be genetically identical. Thus, high-throughput sequencing enables the study of intra-host variation by detecting Intra-Patient Minor Allelic Variants (IPMAVs). These low-prevalence alleles might arise from either single-event mutations in the viral RNA genome occurring within a patient or due to coinfection events (in which case a complete extra set of variant-associated mutations is detected).

For over a decade, the identification of viral quasispecies in infected patients, to characterize genomic variants within them, has been explored for Human Immunodeficiency Virus, Foot-and-Mouth Disease Virus and older SARS-CoV viruses, with improving methodology that has shifted from early clones of PCR fragments to high-throughput sequencing to detect low-frequency intra-patient genomic variants [6,8]. For this purpose, bioinformatic tools such as ShoRAH [9] and PredictHaplo [10] paved the way for the large-scale detection of viral haplotypes in the early days of high-thoughput sequencing, relying on calculating the probability of specific profile sets in assemblies or mapped reads derived from shotgun data. More recent approaches include CliqueSNV, which relies on finding linked polymorphisms based on genomic co-localization, and resolving them using clique selection based on graph theory [11].

Most of the few specialized works currently available on SARS-CoV-2 coinfections focus on particular cohorts of patients during restricted periods and these rely on identifying unexpected mutational profiles belonging to secondary variant haplotypes [12,14]. Coinfections are particularly relevant in the study of SARS-CoV-2 as their occurrence is mandatory for recombination, producing a viable progeny of viruses bearing chimaeric genomes. Effectively, this process represents an expedited evolutionary leap as multiple mutations from the parental variants might get incorporated into the chimaeric genome in a single step and, in tandem, some combinations may prove advantageous, as was the case with currently dominating XBB variants [15].

In the current study, sequencing sets from CoViGen-Mex genomic surveillance, accounting for over one-third of all sequences in Mexico during the COVID-19 pandemic’s emergency phase, were screened for low-frequency mutations that could expose the prevalence of putative coinfections occurring throughout 3 years of the COVID-19 pandemic.

## Methods

### Sample collection and sequencing

This study was a collaboration of Official Public Health Institutions and Institutes forming the Mexican Consortium for Genomic Surveillance (CoViGen-Mex). Clinical procedures for sample collection, diagnosis and storage were designed according to the Mexican Official Norm NOM-017-SSA2-2012 from the Ministry of Health (SSA) [16]. According to the norm, informed consent was waived. Available clinical data was anonymized prior to analysis.

Oropharyngeal or nasopharyngeal swabs were collected between 18 March 2020 and 27 January 2023 in all 32 States of Mexico following the Official Mexican COVID-19 containment programme established by the SSA. The collection was carried out in public laboratories and hospitals from the Mexican Institute for Social Security (IMSS), the National Institute for Respiratory Disease (INER), and the National Cardiology Institute (INC), following the official protocols for sample storage and shipping.

All protocols followed international WHO guidelines and were validated by the Institute of Epidemiological Diagnosis and Reference (InDRE). Briefly, following RNA extraction, RT-qPCR was carried out with 5 µl RNA for a 25 -µl reaction using Superscript III one-step RT-PCR kits (Invitrogen, Darmstadt, Germany). Tubes were incubated for 10 min at 55 °C, followed by PCR for 45 cycles, 95 °C for 15 s, and 58 °C for 30 s. Samples with C_T_ ≤25 were considered for the study.

High-throughput amplicon sequencing tiling the complete viral genome and sequencing was carried out in CoViGen-Mex laboratories (UUSMB-LANGEBIO-CIAD Mazatlan) weekly (except every fourth week) using Illumina COVIDseq library construction kit with primers based on ARTIC v3-v4.1 protocols [5] or a long-amplicon based protocol modified from Castillo *et al*. [17]. Standard Illumina 300 cycles paired-end sequencing (2×150 bp) was carried out following the manufacturer’s instructions for the NextSeq 500/550 or MiniSeq platforms.

### Read processing and variant calling

All genomic datasets were first processed with the standard bioinformatic workflow for genomic surveillance sets in CoViGen-Mex as shown in Fig. S1 to get a variant assignation. For each sample, reads were trimmed to >20 quality ends, and those with short length (<40 nt), low-complexity (<30 %), or with ≥25 Ns were filtered using Fastp v0.19 [18]. Sets were dereplicated with CD-HIT-DUP v.4.6.8, allowing non-matched length and zero mismatches [19]. Reads were mapped against the Wuhan-Hu-1 (NC_045512.2) reference using Bowtie2 v2.3.4.3 [20] with very-sensitive-local, 15 substring length, no soft-clipping, 1000 maximum length and min alignment score of G,30,8. Samtools v0.1.18 [21] was then used for converting to bam, sorting, and indexing. Allelic variant calling was done with its mpileup algorithm (including orphans, no max depth filters, and ≥20 min base quality). Finally, consensus sequences were obtained with the VCF files using a majority rule (≥0.5 frequency) per nucleotide position to improve base-calling. Ns were placed in positions with <20× coverage. Only full-length genomes were used, and those with >10 % Ns were removed from downstream analyses. Datasets passing filters were used for SARS-CoV-2 variant calling using offline Pangolin v4.2 [1]. The GISAID accession numbers of all genomes produced by CoViGen-Mex and collaborators are provided under EPI_SET ID: EPI_SET_231031gz with doi: 10.55876/gis8.231031gz.

The present study uses a dual system for variant classification: first, the Pango nomenclature described above (the actual variant), which is based on the consensus sequences, and a second broader classification including variants of concern (VOCs) such as Alpha, Gamma, Delta and Omicron and variants under monitoring (VUMs). VOC and VUM definitions in this study predate a major simplification by the WHO carried out on 15 March 2023 [22]. Groups of all recombinants and ‘Other variants’ are also included (only B.1.1.222 was kept independent as it was a local variant of interest) and the Omicron VOC is shown as split by their main subgroups: BA.1, BA.2, BA.4 and BA.5. The group-based classification is referred to as VOC/VUM.

Specifically for this study, the resulting VCFs were summarized into condensed allele tables using iVar v1.3.1 [23] with quality ≥20 and ≥0.03 frequency for alleles. This was a critical step as different mutations can co-occur within a sample in the same genomic positions (hence the use of alleles). Original sequences were uploaded to the NCBI’s SRA database under Bioproject PRJNA1008275 and Accession numbers: PRJNA1008275–SRR25735865.

### Epidemiological data

Official figures relating to COVID-19-positive cases in Mexico were obtained through the public repository from the Governmental Directorate on Epidemiology [2] (accessed on 3 March 2023). Date delimiters for epidemiological surges were determined by calculating the lowest number of daily average cases (7 days, step of one) observed during interwave periods and detecting where the curve intersects that mean. This was calculated separately for waves 1–4 (the first delimiter was set to the lowest non-holiday record as there was no inter-wave period) and for waves 5–6 as a lower baseline was required for the latter.

### Alternative allele (mutation) classification

In-house bash v5.1.16(1) and R v4.2.3 [24] code written for downstream analyses is available at https://github.com/rodrigogarlop/IPMAVs for reproducibility purposes under a GNU GPLv3 license. A general workflow is shown in Fig. S2. In summary, an ivar table was created for each sample, containing a complete list of allelic variants per position. Table entries were re-encoded to keep track of the nucleotide position and frequency, the original nucleotide present in the Wuhan-Hu-1 reference, the alternative allele (henceforth, mutations), the sequencing depth, and the strand distribution. Positions having <5 mutations across the complete set of genomes were not considered in downstream analyses.

To reduce spurious observations, mutations mapping a single strand, having <20× coverage, or <0.5 frequency per position were removed. Only alleles present in at least five genomes were preserved. With these data, a contingency table (allele map) was compiled, reporting the allelic frequency per position and genome in the surveillance set. This table was the input for coinfection and mutation analyses. In-house R scripts were used to carry out the statistical analysis of IPMAV and major alleles’ distributions.

### Coinfection and mutation analyses

Two different sets of analyses were carried out using the allele map: first, for mutation analyses, each allele was studied longitudinally in the complete set. These were classified into those that were only observed as IPMAVs (<0.5 frequency in a position in a sample), those only observed as major mutations (either single nucleotide polymorphisms [SNPs] or indels) and present in the consensus (≥0.5), and those that had been detected as both IPMAVs and major in different genomes. Alleles were also collated by position and their type of mutation (SNPs or indels) to assess mutation density. Finally, distributions of total alleles per genome were compared between IPMAVs and major mutations.

Coinfection analyses relied on three main aspects that had to be resolved before exploring the genomes individually: A) identifying variant-defining key mutation constellations (haplotypes), B) detecting mutations that are shared among different variants (base-line noise), and C) determining the time windows in which each pair of variants may have co-circulated (co-circulation timeline).

- Each variant is expected to have a specific constellation of mutations, i.e. key mutations forming a haplotype that varies longitudinally and regionally. A haplotype was created for each variant and VOC/VUM group. Only those with ≥20 observations were considered, except for B.1.631 (16 observations), which is a parental variant for local recombinant XB, and therefore considered of interest in this analysis. Contrary to the regular haplotypes that are commonly reported, mutations present as IPMAVs were included in this analysis. The collection of key mutations per variant was selected by their total prevalence in each group (≥0.7 for variants, and ≥0.6 for VOCs/VUMs). This produced a list of haplotypes for each variant or VOC/VUM group in the study set was expected to bear.
- As variants are the product of an ongoing evolutionary process, different variants might share common mutations either due to shared inheritance, convergence, or recombination. To integrate this information, a map of the pairwise mutation intersections was created for variants and for VOCs/VUMs based on their haplotypes (i.e. which mutations are expected to be shared and thus, non-informative for coinfections). This was turned into a matrix displaying what percentage of each variant is expected to be shared with each other variant/group. Hence, this produced a file bearing the baseline noise of variant assignation.
- Coinfection analyses were also made time-aware to weight the probability of two variants coexisting in any given period. CoViGen-Mex issued a periodic cumulative report on circulating variants in the country, including all genomes available in Mexico [25], with sample collection dates and the assigned variants. This data was accessed on 3 March 2023, to determine putative co-occurrence periods between each pair of variants detected in Mexico. Lone cases of variants re-emerging 2 months after no such variants had been reported were discarded as outliers in the date range calculation, along with those few appearing 2 months ahead of most cases for a variant. However, there were some specific variants that thrived in two separate periods that were set 2 months apart (bimodal, having two separate peaks). To avoid discarding those cases as outliers, density cutoffs were set. Early cases were kept when the rest of the distribution accounted for at least 40 % of the cases, whereas late cases were kept if the rest of the distribution was comprised of at least 60 % cases. Each period was then extended 1 week before and after their first and last reported instances, respectively. This was summarized in co-circulation timeline to determine which pairs may have co-circulated based on their collection date.

For the actual coinfection analysis, each sample had been assigned a variant (or VOC/VUM) based on its consensus genome and, thus, had a predictable collection of key mutations that should be present (expected mutations). In summary, regardless of their frequency within each sample, the actual observed mutations were compared to each target haplotype to evaluate their completeness in that sample (e.g. how complete was an Alpha or Delta profile per genome), whilst ignoring the baseline noise in variant assignation (the correlation of each completeness vector and the base-line noise should not be larger than Pearson’s ρ≥0.99 for VOCs/VUMs and ρ≥0.97 for variants) and those of them co-occurring outside the expected periods. These cases were considered for detailed analyses.

Absolute differences between observed and expected completeness were calculated, and for each sample, variants having ≥50 % of the expected key mutations and >0.5 standard deviation in their completeness (difference ≥ µ±σ) were further analysed. These differences may signal the presence of a secondary profile, possibly belonging to a secondary variant (putative coinfection). In general, samples with a putative secondary variant have different frequency profiles for each variant involved. The primary variant (typically matching the assigned variant for that sample) is commonly comprised entirely of major mutations, whereas secondary variants are commonly observed as IPMAVs (<0.5 frequencies), except for those mutations that match the main variant. Thus, mutations not unique to secondary variants were ignored for further calculations. Out of the remaining differential mutations, completeness was recalculated. The sample was reported to have a coinfection when most mutations share a similar low frequency, and the remaining profiles are still >50 % complete. To study samples longitudinally, primary and secondary variants in coinfections were separated: as primary variants are coded in the genome consensus, the Pango assignation was used. The mutation profiles of secondary variants were compared with each sample’s primary variant. Shared mutations were filtered out, and completeness based on the remainder profile was calculated. Monthly distributions of primary and secondary variants were calculated, with the latter including all alternative variants detected per sample. Comparisons between the underlying genomic distributions and the coinfection distributions based on epidemiological data were carried out with non-parametric Spearman Rank correlations and rank sum Wilcoxon tests.

### Recombinant analyses

Samples bearing putative coinfections were further analysed to confirm or discard signals of recombination. For this, those having at least two major alleles that might not belong to the primary variant were selected, assuming some of the secondary’s variant mutations may have become dominant due to recombination.

A reference dataset was built by filtering Nextstrain’s North America all-time genomic subsampling [26] to keep at most two non-recombinant reference genomes per variant and adding two of each primary or secondary variants (from our set) that might have been missing. Variants XB and XBB descendants were kept as an exception as they had been found in our set as primary variants and the latter accounted for a large fraction of 2022–2023’s references. The target and reference samples’ genomes were aligned and variants were identified using the Nextclade pipeline [27]. These were then subjected to the Recombination Detection Programme (RDP5) [28]. This approach uses multiple recombinant search pipelines to cross validate results based on the recombination signals found by different breakpoint detection methods using an aligned reference dataset to detect parental variants of putative recombinant. Reference EPI_ISL_582311, an A.1 sequence, was set as root, fixed window size to 60 for all adjustable algorithms and ran recombinants analyses using six different methods: RDP, GENECONV, CHimaera, MaxChi, SiScan, and the 3Seq. Only results that were significant at an alpha=0.05 were considered.

## Results

### Genome and variant distribution

**Fig. 1.**
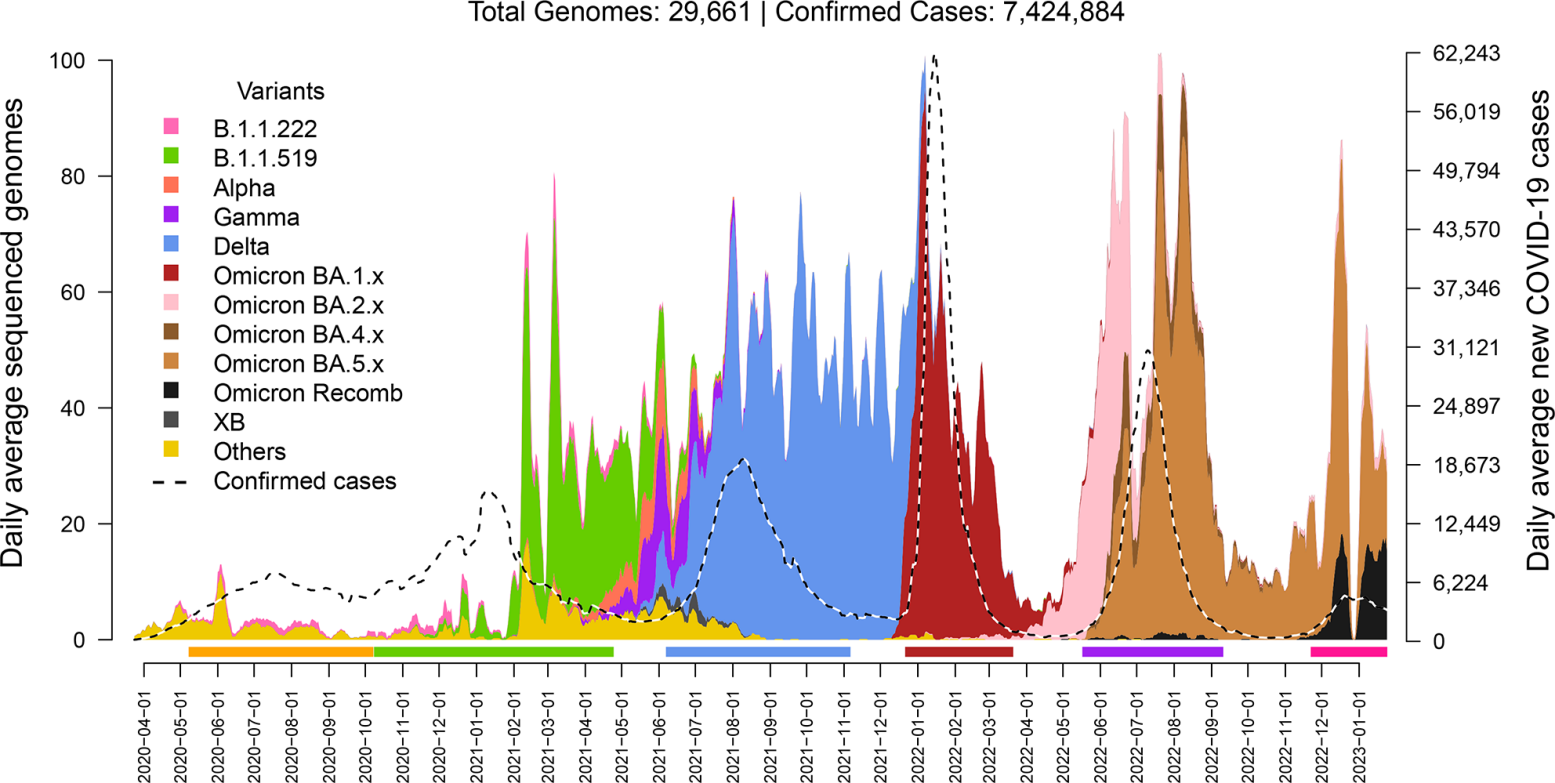
Daily average of sequenced genomes produced by CoViGen-Mex and new COVID-19 positive cases in Mexico. The stacked area plots depict the distribution of novel daily genomes sequenced (scale in left y-axis) based on their collection date and coloured by variant group. The dashed line shows the daily positive COVID-19 cases in the official Mexican epidemiological data (scale in the right y-axis). Both sets were reported by collection date and are presented in a sliding 7 day average window with a step of 1 day. Areas are stacked, having no group overlap. Epidemiological waves marked with bars at the bottom are defined as follows: Wave 1 (5 May – 4 October 2020; peak on 18 July 2020 [7 275.29 average daily cases]). Wave 2 (5 October 2020 – 27 April 2021; peak on 10 January 2021 [16 010.00 cases]). Wave 3 (11 June – 4 November 2021; peak on 10 August 2021 [19 305.86 cases]). Wave 4 (23 December 2021 – 22 March 2022; peak on 15 January 2022 [62 134.71 cases]). Wave 5 (15 May 2022 – 6 September 2022; peak on 10 July 2022 [30 824.43 cases]). Wave 6 (22 November 2022 – 27 January 2023; peak on 20 December 2022 [4 850.00 cases]).

A total of 29 661 SARS-CoV-2-positive samples collected from 18 March 2020 to 27 January 2023, in different collaborating clinics and hospitals across the country, produced high-quality genomic sets. The longitudinal sample distribution of this dataset is observed in Fig. 1 and compared to the epidemiological data (dashed line). Clinical and genomic data are reported in Table S1. As can be observed in the figure, the pace of sequencing increased starting in February 2021, following the standardization of the monthly surveillance protocols.

According to the General Epidemiology Directorate (DGE in Spanish), as of 28 June 2023, 7 424 884 COVID-positive cases had been confirmed in Mexico, distributed in six epidemiological waves (Fig. 1). Waves 3–6 coincided with the onset of new VOCs (e.g. Delta, Omicron and their subvariants) and other successful variants (e.g. the VUM B.1.1.519).

The first wave was characterized by a seemingly even competition between variants, none of which bore clear dominance. Variant B.1.1.222 was the first one to become ubiquitous in the country by October 2020 (up to >60 % of the daily average cases), followed by B.1.1.519, which dominated the second wave (up to ~90 % of all cases). The inter-wave period between the second and third waves saw the onset of the first VOCs) in the country in spring 2021. Of these, Alpha, (~22 % of all cases at its highest), which was most frequent in the northwest, while Gamma (~35 %), was mostly detected in the southeast. The large variant diversity would swiftly dwindle after the expansion of the Delta VOC and its subvariants, which by August 2021 had virtually displaced all others. Similarly, the arrival of VOC Omicron (BA.1) in December 2022 meant the end of Delta’s dominance as it started a new phase of the pandemic, in which Omicron-derived subvariants succeeded one another, with full dominance of Omicron BA.1 during the fourth wave, BA.2 variants dominating the following inter-wave period, and BA.5 dominating the fifth one. The sixth wave has additionally seen the rise of recombinant Omicron variant XBB (accounting for >50 % of daily average cases by the end of the study).

A total of 372 different variants were identified in the present set, 301 (93.12 %) of which were VOC, VUM, or recombinants.

### Mutation analysis

Based on the reference genome, mutations were found in 28 607 different positions (ignoring singletons). The 1 866 753 mutations in the entire dataset were classified into 25 053 different alleles with an associated per-position frequency.

In total, 14 973 mutations were observed in at least five different genomes, 534 and 133 of which were insertions and deletions, respectively. The rest corresponded to point mutations (39.35 % were non-synonymous).

**Fig. 2.**
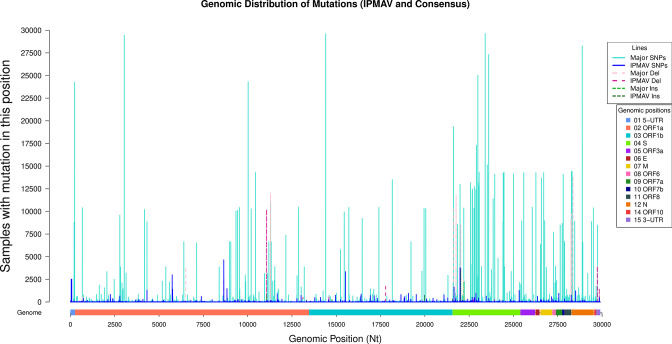
Genomic distribution of all IPMAVs and major mutations in the CoViGen-Mex set. The height of each line (y-axis) represents the total samples that have a mutation in a specific position in the SARS-CoV-2 genome (x-axis). Major mutations are shown in light colours and IPMAVs are in dark colours. SNPs are shown in shades of blue and continuous lines, deletions in pink/red and dashed lines, and insertions in green on dotted lines. The x-axis shows the genomic map (including genes, UTRs and ORFs).

Despite mutations being widely distributed among the different genomic positions, mutation density was far higher in the S gene (Fig. 2), with 2623 different mutations in 3.82 kb (686.64 mutations per kb) compared to 6304 mutations in 13.20 kb of ORF1a (477.58 per kb) and 2781 mutations in 8.09 kb in ORF1b (343.76 per kb).

**Fig. 3.**
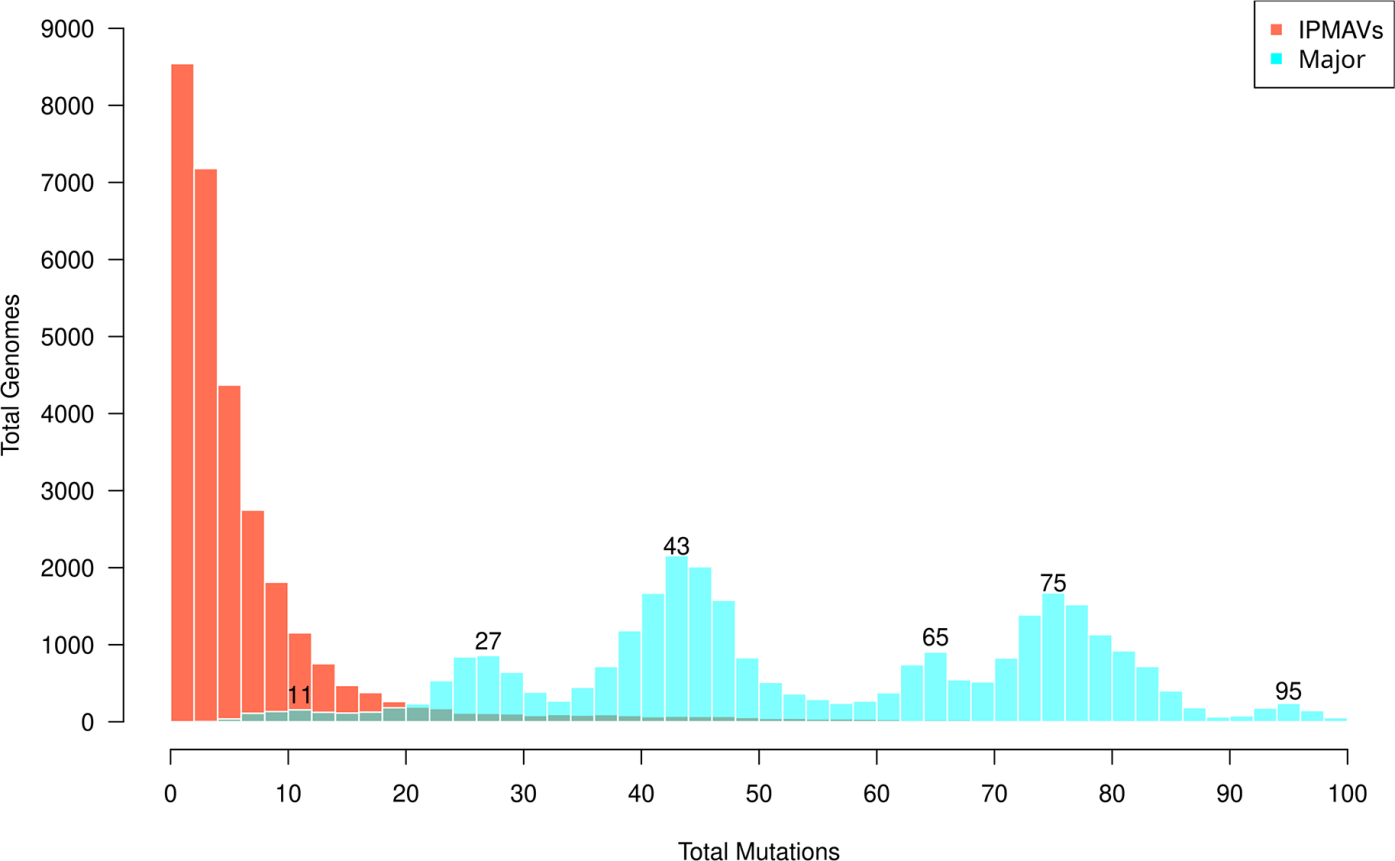
Distributions of total IPMAVs and major mutations per genome. Overlapping histograms show the total genomes (y-axis) having each number of mutations (x-axis). For major mutations, the five most relevant local modes are shown.

Intra-Patient Minor Allelic Variants (IPMAVs), the main focus of this study, were defined as mutations occurring within a sample with a frequency of <0.5 at any given loci (i.e. the majority of reads in that position do not bear that specific mutation). IPMAVs are usually ignored when constructing a consensus as this only considers major mutations (those with frequency ≥0.5 in this study). IPMAVs were found in 28 623 samples (96.50 %), although the total number of IPMAVs detected per sample followed a power law distribution, with 18 170 samples (61.26 %) having five or fewer IPMAVs among their mutation collections (Fig. 3). Contrastingly, the total major mutations per genome had a drastically different multimodal distribution (mean=54.82±20.50) with modes at 11, 27, 43, 65, 75, and 95 major mutations (shown in the figure) that reflect the statistically discrete but progressive accumulation of mutations occurring by the onset of different VOCs and VUMs that were widely distributed in Mexico. More specifically, 32.39 % of the samples bearing 10–19 major mutations were B.1.1.222; 77.38 % with 20–29 were B.1.1.519; 79.91 % having 40–49 were Delta subvariants; 78.79 % with 60–70 were subvariants of Omicron BA.1; 66.15 % of those with 70–80 were BA.5 and 25.43 % BA.2; and 81.40 % with 90–100 mutations are XBB Omicron recombinants.

In total, 14 973 mutations were observed at least once as IPMAVs (95.87 %) and only 618 (4.13 %) were observed exclusively as major mutation. Interestingly, 3881 mutations were detected exclusively as IPMAVs, meaning they were never observed as part of any genome consensus from a sample, although only 383 of those were present in ≥20 samples (Table S2). Despite these differences, the gene distribution of IPMAVs strongly correlated (ρ=0.94) with that of major mutations and it cannot be discarded that both distributions had been drawn from the same population (Wilcoxon rank sum test *p*-value=0.68).

### Key mutations and coinfection analysis

A total of 104 different key mutation profiles (haplotypes) were calculated from all Mexican samples, one for each variant having ≥20 genomes (Table S3). These ranged between five and 96 key mutations per haplotype. A second set of profiles was created from the mutation distributions in the nine most prevalent groups of variants that circulated in Mexico (henceforth VOCs/VUMs/recomb), including B.1.1.222, B.1.1.519, Alpha, Delta, Omicron subgroups BA.1, BA.2, BA.4, BA.5, and recombinants XB, XBB, and XAS (Table S4). Their total key mutations ranged between four and 91 per haplotype.

The completeness of each of the 104 haplotypes was tested for every sample in the dataset based on which of the expected mutations were present. Predictably, the variant profile assigned to the consensus genome was usually found with completeness nearing 100 %. Also, all variants belonging to the same group (e.g. Delta) shared a higher number of mutations, and thus, if present in a sample, resulted in multiple high-completeness variants per sample (e.g. presence of any of the 14 different B.1.617.2.x profiles [Delta], abbreviated AY.x, would result in multiple other high-completeness Delta profiles). Redundancy among variants was accounted for by filtering shared mutations and variants that did not co-occur in the same periods for downstream analyses.

VOCs/VUMs/recomb (including B.1.1.222) collectively account for most samples in the set (94.77 %), with VOCs accounting for 82.50 %. Considering only VOCs (Alpha and Gamma, Delta and Omicron), those detected with a secondary profile with 50 % completeness were analysed (in all cases, primary variants are those identified with the consensus using Pango). A total of 59 (0.21 %) samples had haplotypes involving more than one VOC. These cross-VOC putative coinfections were used as a proof of concept for downstream variant-scale analyses as the involved VOCs had largely different haplotypes. Most importantly, their analysis revealed that the secondary variant was comprised of low-frequency IPMAVs (Table S5), except in four cases, meaning the main variant was indeed part of the consensus. However, the secondary one had been detected in a lower proportion. Out of the secondary variants, 17 were Alpha, 13 were Gamma, 30 were Delta, and one was Omicron. Four of them had two secondary variants, and 31 samples were from the early-VOC and Delta periods, whereas 24 were post-Omicron.

For the complete set of 104 variants (Table S6), the search was targeted toward mutations in secondary variants that A) were mostly seen as IPMAVs and B) were not part of the primary variant. The analysis resulted in the detection of 379 putative coinfection cases in the current data involving at least one secondary variant for a total prevalence of 1.28 % in the entire set.

A demonstration of a single putative coinfection case is provided as an example in Fig. S3. Genome EPI_ISL_2801846, from June 2021, was classified as variant B.1.1.28.1 (Gamma). A putative coinfection was identified as a composite of two haplotypes in the same genomic dataset. The primary variant was confirmed with a full B.1.1.28.1 profile, where all 36 key mutations were present, 35 of which were major mutations (completeness was 100%). The secondary profile matched variant B.1.1.7 (Alpha) with 33 out of 33 mutations present. Ten mutations were shared between both variants. This leaves 23 unique expected mutations in the Alpha profile, all present in the sample. More importantly, secondary mutations were all IPMAVs with mean frequencies=0.17±0.06, while mutations unique to Gamma were all in the consensus with mean 0.73±0.15. This suggests the coinfection may have occurred in a proportion of three Gamma viruses per one Alpha virus.

**Fig. 4.**
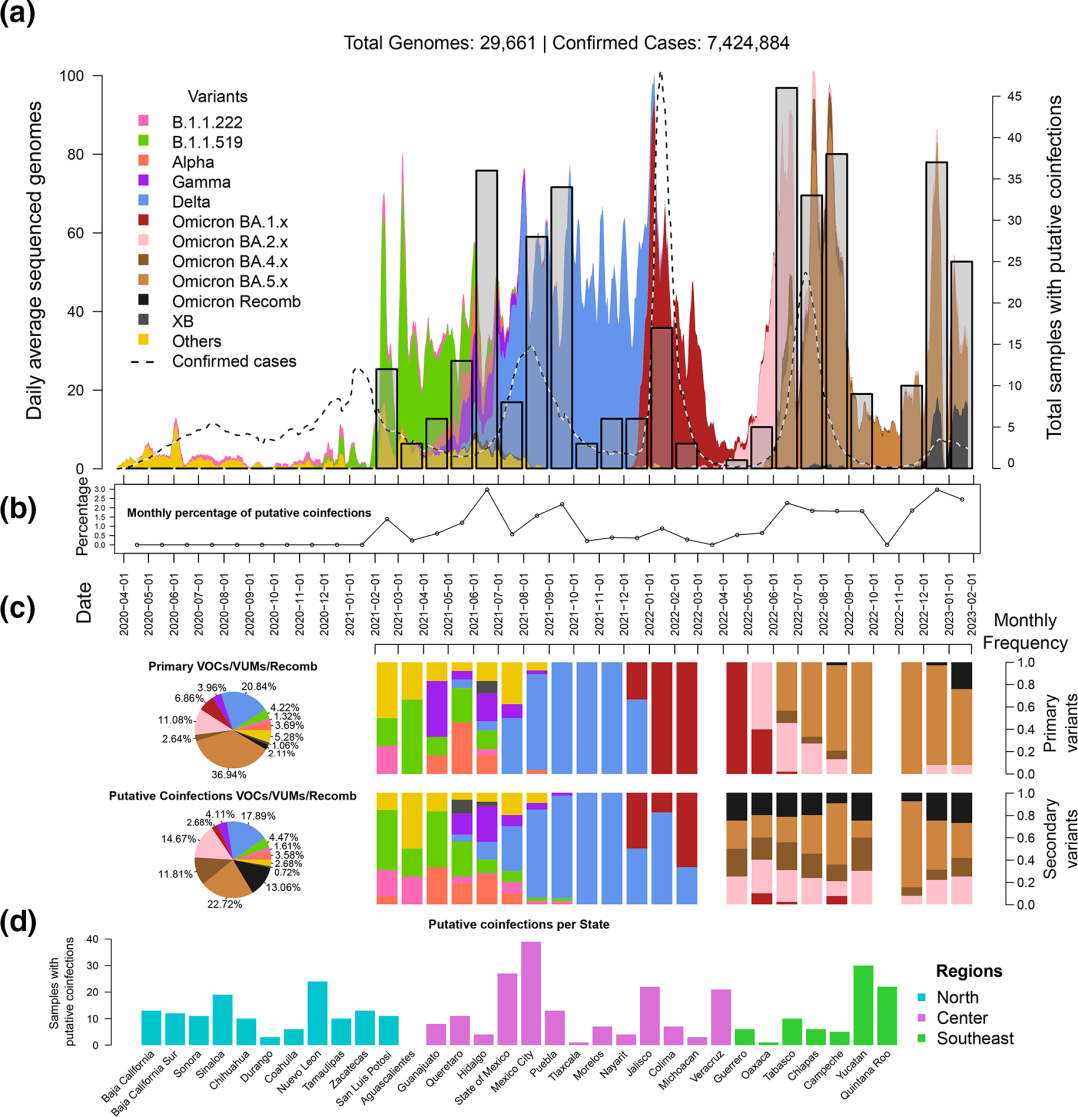
Description of putative coinfections detected in CoViGen-Mex’s data. All longitudinal data is shown using the same periods (x-axis). (a): The monthly total coinfections (grey transparent bars) are shown over the distribution of samples shown in Fig. 1 (starting on the first of each month and ending on the last day of the month). (b): The black line with circle bullets shows the monthly percentage of detected coinfections out of the total samples available for each month. (c): On top, the collated primary haplotype frequency, by VOCs/VUMs/Recombinant group in all putative coinfections (pie) and their monthly frequency (bars). Similarly, the pie below shows the secondary haplotype collated prevalence, and their monthly frequency (bars). (d): The geographical distribution is shown per state, with the y-axis showing total putative coinfections detected in all sets and coloured by region.

Based on the analysis of all 379 putative coinfections, samples containing admixed profiles mirrored the distribution of the monthly sequenced genomes in the study (Fig. 4a). Their distributions bore no significant statistical differences (*p*-value 0.42). Plus, there was a strong correlation between them (ρ=0.84), supporting that total coinfections detected are proportional to sequenced genomes. The percentage of cases represented by coinfections varied monthly (Fig. 4a), only observed from February 2021 onwards, with a mean of 1.32±0.90 % considering only months where they were detected, and peaks in June 2021 (2.98 %), June 2022 (2.26 %), and December 2022 (also 2.98 %). Most of the putative coinfections (59.63 %) were identified in samples where the primary variant was from the Omicron group (subgroup BA.5 accounted for 36.94 %, see Fig. 4b), whereas for 20.84 %, it was Delta. This also mirrors the variant distribution of the whole sample set (48.13 % Omicron and 30.06 % Delta).

The distribution of primary variants in putative coinfections varied according to the variant succession in Mexico. January to July 2021 was the most diverse period, marked by the presence of B.1.1.519, Alpha and Gamma primary variants. June 2021 showed the largest variant diversity, having a record 36 putative coinfection cases (2.98 %), involving variant B.1.1.519 (its monthly prevalence among all samples was 10.75 %), VOCs Alpha (15.63 %), Gamma (29.94 %) and Delta (24.00 %), and the XB recombinant (4.88 %). In the figure, the legend ‘Others’ encompasses variants like B.1.2, B.1.432, B.1.621, B.1.631, B.1.632, B.1.634, B.1.635, and C.37 (former variant of interest Lambda). Of these, B.1.631 and B.1.634 were especially interesting as these two are the parental variants of recombinant XB, the earliest to circulate in Mexico, but their secondary variants were other VOCs. By August 2021, the peak of the third wave, most primary variants were Delta. The peak of coinfections for this wave was registered on September, with 34 putative coinfections (2.19 %), most of them of subvariants of Delta.

Starting in January 2022, all coinfections had Omicron primary variants, BA.1 during the fourth wave (late 2021–early 2022), BA.2 starting in May 2022, and BA.5 starting in June. Although January 2022 registered the largest number of positive cases throughout the pandemic (fourth wave), coinfections were minimal as BA.1 had a clear dominance in the period. During the second semester of 2022, a new increase was observed on par with the total genomes that were sequenced during the fifth wave, spanning June (2.26 %), July (1.84 %), and August (1.82 %), and mostly comprised of Omicron BA.2 and its BA.4/BA.5 descendants. The last period on record having a higher prevalence of putative coinfections was during the sixth wave, in December, when 37 cases were detected over 1243 samples (2.98 %), when subvariants from the BQ and BW (Omicron) lineages were dominant and followed by the arrival of recombinant XBB (Omicron as well). Regarding recombinants, only one putative coinfection was detected with recombinant XAS as its primary variant, (August 2022), and during the last 2 months of the study (December 2022, January 2023) coinfections had XBB variants as the primary variants.

Mutations from secondary variants typically occurred at a much lower frequency than that of the primary variants, possibly reflecting the uneven distributions of the virions bearing each type of genome within a patient. By calculating the median frequency for each profile separately in the primary variant, their average in all samples with coinfection was 0.94±0.11 whereas in secondary variants, the analogous mean per sample was 0.23±0.20, showing a larger variation. It should be noted that the analysis was tailored for detecting coinfections as VAMIPs specifically. Haplotypes were mostly complete for both variants in the coinfections, with primary ones having an average of 98.06±4.25% completeness and 90.89±9.16 % for secondary variants, or 75.40±13.13% when redundant mutations were not considered.

At the finer level of single variants, 75.73 % of the putative coinfections had multiple alternative possibilities for the secondary variant (the full set had median=4, IQR=9). However, detecting admixed variant profiles does not imply that more than one secondary variant was present in a sample, but that its identity could not be confidently established (all alternatives were preserved in the results for analytic purposes). This situation was most commonly observed whenever secondary coinfections were from the same VOC as the primary variant, as seen in most samples with either Delta or Omicron (72.30 %). Furthermore, 68 out of 78 samples with Delta primary variants had another Delta subvariant as their secondary variant (coinfection occurred in-group), whereas 206 out of 217 samples had Omicron were in-group coinfections. The highest number of putative coinfection alternatives per sample was observed for Omicron at 45 whereas for Delta it was 16, which reflects their respective within-group variability in the set. However, if secondary variants are grouped together in larger VOC/VUM groups, 92.61 % of the samples had only one group of secondary variants. The most common combinations of cross-VOCs/VUMs were Delta primary with either Alpha, Gamma or B.1.1.222/B.1.1.519 (B.1.1.519 has all mutations in B.1.1.222 plus 11 other ones). In total, 569 possible different secondary variants were evaluated.

Secondary variants in putative coinfections were distributed similarly to primary variants among VOCs/VUMs/recomb (Fig. 4c), especially in the pre-Omicron months. Globally, the major difference was that BA.5 secondary variants accounted for a lower percentage (22.72 %) than in primary variants, while BA.2 and the Omicron recombinants were more prevalent in the secondary variants (11.81 and 13.06 %, respectively). The monthly distribution also varied, with B.1.1.519, Alpha, and Gamma recovered up to September 2021, albeit in scarce quantities. Delta also prolonged its appearance as trailing Delta secondary variants were observed in February 2023, over a month after Omicron replaced Delta. Contrary to the primary variant distribution and the general distribution in the genome set, BA.2 and its descendants (BA.4/5 and XAS/XBB) are reported earlier than expected among the secondary variants, starting on 31 April 2022 and this continued to be detected throughout all the post-Omicron period (e.g. XAS recombinants from April to September 2022 or XBB in the last 3 months). This may show that the method’s resolution is possibly suboptimal for Omicron, precisely due to the high homogeneity of the haplotypes of BA.2 and descendants as they are all phylogenetically close. Further refining results might be possible through manual curation, but each case must be evaluated separately based on its full context.

The geographical distribution of samples potentially containing coinfections has a very similar distribution to that of all genomes in the set (ρ=0.90), showing no statistically significant difference between both populations (*p*-value=0.88), meaning the impact of the sample distribution is not negligible. Regarding the coinfection distribution, Mexico City and the State of Mexico, which together contain the single largest metropolis in the country, account for 66 putative coinfections (17.41%), a reflection of the genome’s distribution. If analysed by State, as shown in Fig. 4d, 48.13 % of all coinfections were found in the Centre region, the most populated area in the country where Mexico City is located and the one producing the most genomes, with 48.13 % of all reported coinfections, followed by 34.83 % in the Northern region, and the Southeastern regions with 21.11 %. Likewise, the patient status distribution did not show a statistically significant difference when compared to that of the complete set of genomes (*p*-value=0.875), having 67.81 % of coinfection in ambulatory patients, 30.87 % hospitalized, 0.26 % deceased and 1.06 % unreported. The same occurred for the age distribution (*p*-value=0.9658), in which the largest groups were those of persons aged 20–29, 30–39, and 40–49 years old (20.05, 21.11, and 16.09 %, respectively).

The 379 samples bearing putative coinfections were further checked for the presence of recombinants. There were 147 of them in which the secondary variants had at least two major mutations that were not part of the primary variant, meaning they had become part of the consensus genome, a first insight into potential recombinant events. A map of the overlapping mutation profiles is provided for each pairwise permutation and is available at the git repository. Using their genomes and a custom set including references from most variants that have circulated in North America, a multi-algorithm approach was carried out for detection. Still, no cases of recombinant events in the target dataset were identified (>0.05 significance).

## Discussion

Most standard genomic analyses with SARS-CoV-2 are bound to studying only allelic variants that appear as part of consensus sequences, since the focus is epidemiological surveillance. The present study aimed at describing Intra-Patient Minor Allelic Variants (IPMAVs), low-frequency alleles that are not part of the consensus sequences and instead co-occur with less than 0.5 frequency per nucleotide position. IPMAVs are a constant in viral genomic sequencing sets, detected in 28 623 out of 29 661 (96.50 %) genomes analysed in this study. Having produced over 35 % of the SARS-CoV-2 genomic data that has been sequenced in Mexico, CoViGen-Mex’s sample dataset is representative of the known genomic distribution in all 32 States in the country as deposited in GISAID [3], making this the first nationwide catalogue of low-frequency mutations in Mexico. Although this sample concentrates on patients having attended public hospitals where the samples were taken, it has managed to follow the subtle changes in the prevalence of variants over time in the different regions of the country, marked by six epidemiological surges (waves, Fig. 1) and multiple variant turnover events that contribute to the underlying change in the dynamic process.

The importance of studying IPMAVs, as the current manuscript describes, allows the detection of putative coinfection events, which represent a scenario that may propitiate the necessary conditions for genomic recombination. The response to the COVID-19 pandemic has produced an unprecedentedly large volume of genomic sequences that have been generated from samples worldwide (15 868 843 in GISAID on 10 August 2023). In addition, the amplicon-based targeted sequencing that has been commonly used to detect the genome sequence of this virus [5] yields a much more even coverage of the whole genome, enabling the detection of fine differences at the single nucleotide level that are not restricted to a few genes like most viral studies of the past, but spanning the complete genomic landscape, including the non-coding regions. Furthermore, the extensive sampling effort has allowed the scientific community to track the evolutionary process of the SARS-CoV-2’s genome exhaustively at the level of specific mutations [4], and to designate new variant names as the evolutionary paths continue to diverge. All this makes it increasingly plausible to detect not only the major allelic variants that form the consensus, but also mutations that are not part of the expected profiles for a given variant.

IPMAV tracking and its subsequent search for coinfections can be considered by-products of SARS-CoV-2 genomic surveillance obtainable by any institution carrying out genomic sequencing, providing the original sequencing read sets are preserved. However, the technical procedures are non-standard, and thus, they still need to be fully explored. Most available efforts focus on particular cohorts, locations, or periods. For instance, in 2022, Combes *et al.* described seven immunocompromised patients who were infected with both Omicron and Delta variants (first detected with RT-PCR and classified through WGS). These cases accounted for 0.2 % of 3 237 samples [12]. In the same year, Molina-Mora *et al.* published their results from a controlled *in vitro* study consisting in the analysis of mixed samples of different permutations of variants Alpha, Beta, Gamma, Delta and Omicron and other seven non-VOC variants including B.1.1.519 [14]. Even though their method was based on approaches originally designed for bacterial 16S amplicon data, they succeeded in detecting admixed haplotypes to identify coinfections. Most importantly, they concluded that using sequence reads, denoised as amplicon sequence variants (ASVs), was better suited than assembling for coinfection identification, supporting the approach used in the current study. They also reported that the identification of a VOC was possible if at least three ASVs were mapped to a reference VOC genome, a simpler criterion than the one used in the current study but yielding good enough results for detecting cross-VOC coinfections. This group also inferred that detection would require several thousand genomes to detect even a single coinfection as they failed to detect any in 1021 genomes from the first year of the COVID-19 pandemic in Costa Rica.

The overall average prevalence of coinfections in the current set was 1.28 % but varied monthly, ranging from 0.21–2.98 % (Fig. 4), showing no specific distribution due to the patient’s geographical localization, age, or hospitalization status. In 2022, Zhou and collaborators carried one of the largest coinfection studies published to date [13], with 50 809 ultra deep sequencing samples generated in the United States between January 2021 and September 2021. They reported 195 coinfections, a rate of 0.38 %, which is closer to the lower limit in the current study. Contrastingly, another large-scale study from Brazil by Schrörs *et al.* placed their estimate at 2.6 % using 30 806 genomic datasets [29], closer to the upper limit of the current study. Differences between their works and ours were primarily methodological, mainly in the definitions of key mutations and filters used, and in the consideration of synonymous mutations, those in non-coding regions, and IPMAVs for defining the expected haplotypes.

The two most determinant factors for detecting coinfections in the current study were the number of sequences analysed and the variant diversity at each period. This observation partially explains why no coinfections were detected prior to February 2021 as, on average, the period was poorly sampled, with only 87.09±49.26 daily genomes available prior to that month. Precisely considering the monthly observed prevalence of coinfections in the current set, one coinfection would be expected for every 33.60 to 474.33 total genomes that get sequenced (on average, 145.85±131.97) depending on each month’s variant distribution. In addition, prior to B.1.1.519’s expansion in December 2020, no other variant had been as widely distributed and dominant in the national landscape, contrary to all other putative secondary variants that came afterward. The first year has been described as a period when multiple variants competed under similar conditions, before the first vaccines had been widely distributed [30]. This highlights the importance of keeping an adequate surveillance effort, even after the emergency phase of the COVID-19 pandemic has ended.

The first peak in coinfection prevalence was observed during June 2021 (the inter-wave period between waves 2 and 3, see Fig. 4), the period with the highest number of co-occurring VOCs/VUMs to date in Mexico [31]. A second peak was observed during the third wave in September 2021, when only the Delta variant was circulating, albeit displaying a large subvariant diversity dominated by AY.20 and AY.26, as has been described before [32]. Interestingly, average coinfections dropped during the fourth wave, having the largest record of COVID-19 cases in Mexico so far. This may have been due to the swift turnover event of Omicron’s sweep, which superseded Delta almost entirely in only 2 weeks, leaving no chance for any other variant to compete, as has been suggested previously [33]. In the period that followed, coinfections mirrored the diversification of the Omicron variants throughout waves five and six, although only the primary variants managed to capture this more accurately.

Even though no recombination events were found within the putative coinfection set, there are several factors that might explain this observation: first, ongoing recombinations are expected to be rare events, which makes them unlikely to be detectable with only a snapshot of an ongoing coinfection as it also depends on how well the newly created recombinant fares against the parental variants within the same infection. Normally, most recombinants are detected after virions carrying the new chimaeric genome start a new infection and thus have no secondary variants present. Second, the preceding coinfection search was aimed at detecting secondary variants bearing preferentially low-frequency mutations, meaning most of their mutations would not be present in the actual consensus genomes. Forcing them into the consensus proved not to be a viable solution either because secondary profiles in our set were mostly complete and had mutations spanning the whole genome in a way that having both sets of mutations would create an homogeneously mixed chimaera, not clustered sets of mutations as would be expected in recombinants. Third, since the onset of VOCs, there have been several major genomic bottlenecks as the most successful variants tend to outcompete all others, with a few lineages effectively driving each epidemiological wave on their own. This results the detection of fewer mutations that are differentially distributed when the two parental variants come from the same lineage, which further hinders recombinant detection. Since January 2022, only Omicron variants exists in Mexico and the baseline number of shared mutations has increased with each consecutive wave (more recently, XBB-XBB cases have over 100 shared mutations). In this case, mutations from secondary variants in a coinfection would actually represent confounding factors in the recombinant analysis as several contemporary variants are just a few nucleotides apart.

It cannot be discarded that a small percentage of IPMAVs in the current dataset might have arisen from sample contamination, amplification (commercial high fidelity polymerases range from 2.4×10^−6^ to 3×10^−5^ [34]) or sequencing errors (the error rate of SBS sequencing has been reported to be 0.24 ± 0.06 % [35]). Regarding contamination, no access was granted to most sequencing schemes (several laboratories were involved). Thus, looking at sequencing data, coinfections and contamination would appear identical and contamination cannot be ruled out. Sequencing errors were mitigated by considering only alleles present in at least five genomes. However, IPMAVs which appear as isolated peripheric mutations, many of which are transitory, and part of the natural evolutionary process, can be identified. This is reflected in the differences in the distributions of total IPMAV and major mutations per genome (Fig. 3). The number of major mutations was multimodal, with modes representing the progressive accumulation of expected mutations that arose due to the selective pressure as the virus escaped the immune system and improved their transmissibility, ranging from B.1.1.222 (ten key mutations) to XBB (90–100 mutations). IPMAVs, on the other hand, followed a power law distribution where most genomes had fewer than ten of them. Most of these arise probably not from coinfections, but as part of the ongoing evolutionary process within a single patient, where mutations arise by chance and many are lost to genetic drift. Congruent to previous studies [36], both major mutations and IPMAVs occur most commonly in the ORF1a, ORF1b, or S genes but showed no statistically significant differences between their distributions.

Variant-calling for the detection of secondary variants should not depend on single mutations but on collections of multiple specific mutations in a genome, which can be observed as mutation profiles that are inherited and shared by phylogenetically related sequences. In the current study, up to 86 alternative alleles per putative coinfection were observed (up to 42 after filtering mutations redundant to those of the primary variant). Thus, the probability of detecting the exact full haplotypes matching a reported variant by chance alone is greatly reduced and made errors negligible when profiles were mostly complete, as was the case for the 379 samples selected as putative coinfections.

Five limitations in the current study should be highlighted: 1) Mutation profiles of a single variant are not static in real case scenarios. As a result, variant definitions are typically partially flexible, allowing for a few additional or missing mutations that fluctuate from the expected haplotype. Mutations occur constantly, even during the same infection (the error rate is estimated at ~1.3×10^−6^ mutations per replication cycle [37], although the rate of evolution has been reported to be higher in branches that give rise to new VOCs [38]). 2) Variants are not identical across countries (or even regions) and keep on accumulating changes after multiple successive transmissions occur, eventually giving rise to new variants as selective pressure or founder effects result in different subvariants with distinct geographical distributions (e.g. additional mutations in Mexican BA.5.6.2, which eventually gave rise to BW.1 [39]). To cope with these two limitations, mutation profiles (haplotypes) were customized to reflect the precise allelic distribution in the Mexican genomes of these variants. 3) Uncertainty increases for secondary variants when working with those containing fewer key mutations or that are closer genetically. 4) Mutations are shared across variants due to convergence. 5) Sampling was normally biassed to specific States, such as Mexico City and Quintana Roo, depending on the period. Also, sampling in some States is more intermittent than others in regarding available samples, especially during the last waves as the number of samples declined.

As future perspectives, a nationwide exploration of putative recombinant variants through breakpoint discovery would aid in determining if any of the predicted coinfections may have led to recombination events. Also, new samples collected throughout 2023 will be used to identify updated variant profiles and detect additional putative coinfections. As it can be inferred from the first part of the sixth wave in the data, the XBB recombinant variant has an advantage over other circulating variants. The present analysis of coinfections can give us insights into some of the factors that help shape viral diversity and ultimately may impact recombination rates. Also, regardless of which variants dominate the epidemiological landscape, coinfections are expected to continue occurring in low percentages and perhaps they can be useful to monitor mutations that are normally ignored as IPMAVs.

## Conclusions

IPMAV discovery can enable the detection of coinfections by detecting secondary variant-specific haplotypes occurring within the same patient. In most cases, these are observed in a lower proportion compared to the main variant in the putative coinfections, and near-complete mutation profiles can be recovered from the datasets. In Mexico, the prevalence of coinfections was reported to vary longitudinally. It was higher in periods when multiple variants circulated in similar proportions, especially during major turnover events such as the onset of the first VOCs, the diversification of Delta and the first Omicron sweep. The overall prevalence of coinfections observed in Mexico was higher than in most previous reports, but their detection depends on the total genomes available per period and the variant diversity, which varies between countries. The fact that coinfections can lead to recombination highlights the need to keep an adequate surveillance effort in all countries.

## supplementary material

10.1099/mgen.0.001220 Fig. S1.

10.1099/mgen.0.001220 Table S1.

## Abbreviations

CoViGen-Mex: Mexican consortium for genomic surveillance
IPMAV: intra-patient minor allelic variant
PCR: polymerase chain reaction
VOC: variant of concern
VUM: variant under monitoring

## References

[1] Rambaut A, Holmes EC, O’Toole Á, Hill V, McCrone JT (2020). A dynamic nomenclature proposal for SARS-CoV-2 lineages to assist genomic epidemiology. Nat Microbiol.

[2] Secretara de Salud - Gobierno de México (2023). Datos Abiertos Dirección General de Epidemiología Secretaría de Salud Gobierno. https://www.gob.mx/salud/documentos/datos-abiertos-152127.

[3] Khare S, Gurry C, Freitas L, Schultz MB, Bach G (2021). GISAID’s role in pandemic response. China CDC Wkly.

[4] Chen C, Nadeau S, Yared M, Voinov P, Xie N (2022). CoV-Spectrum: analysis of globally shared SARS-CoV-2 data to identify and characterize new variants. Bioinformatics.

[5] Farr B, Rajan D, Betteridge E, Shirley L, Quail M (2022). COVID-19 ARTIC v4.1 Illumina Library Construction and Sequencing Protocol-Tailed Method V.2 DNA Pipelines R&D, Protocol Citation: DNA Pipelines R&D. Epub Ahead of Print.

[6] Ode H, Matsuda M, Matsuoka K, Hachiya A, Hattori J (2015). Quasispecies analyses of the HIV-1 near-full-length genome With Illumina MiSeq. Front Microbiol.

[7] Cacciabue M, Currá A, Carrillo E, König G, Gismondi MI (2020). A beginner’s guide for FMDV quasispecies analysis: sub-consensus variant detection and haplotype reconstruction using next-generation sequencing. Brief Bioinform.

[8] Xu D, Zhang Z, Wang F-S (2004). SARS-associated coronavirus quasispecies in individual patients. N Engl J Med.

[9] Zagordi O, Bhattacharya A, Eriksson N, Beerenwinkel N (2011). ShoRAH: estimating the genetic diversity of a mixed sample from next-generation sequencing data. BMC Bioinformatics.

[10] Prabhakaran S, Rey M, Zagordi O, Beerenwinkel N, Roth V (2014). HIV haplotype inference using a propagating dirichlet process mixture model. IEEE/ACM Trans Comput Biol and Bioinf.

[11] Knyazev S, Tsyvina V, Shankar A, Melnyk A, Artyomenko A (2021). Accurate assembly of minority viral haplotypes from next-generation sequencing through efficient noise reduction. Nucleic Acids Res.

[12] Combes P, Bisseux M, Bal A, Marin P, Latour J (2022). Evidence of co-infections during Delta and Omicron SARS-CoV-2 variants co-circulation through prospective screening and sequencing. Clin Microbiol Infect.

[13] Zhou H-Y, Cheng Y-X, Xu L, Li J-Y, Tao C-Y (2022). Genomic evidence for divergent co-infections of co-circulating SARS-CoV-2 lineages. Comput Struct Biotechnol J.

[14] Molina-Mora JA, Cordero-Laurent E, Calderón-Osorno M, Chacón-Ramírez E, Duarte-Martínez F (2022). Metagenomic pipeline for identifying co-infections among distinct SARS-CoV-2 variants of concern: study cases from Alpha to Omicron. Sci Rep.

[15] World Health Organization (2023). WHO Risk assessment of XBB.1.5. https://www.who.int/docs/default-source/coronaviruse/20230620xbb.1.5.pdf?sfvrsn=fff6f686_3.

[16] Gobierno de México (2013). DOF - NOM-017-SSA2-2012, Para la vigilancia epidemiológica. https://dof.gob.mx/nota_detalle.php?codigo=5288225&fecha.

[17] Castillo AE, Parra B, Tapia P, Acevedo A, Lagos J (2020). Phylogenetic analysis of the first four SARS-CoV-2 cases in Chile. J Med Virol.

[18] Chen S, Zhou Y, Chen Y, Gu J (2018). fastp: an ultra-fast all-in-one FASTQ preprocessor. Bioinformatics.

[19] Fu L, Niu B, Zhu Z, Wu S, Li W (2012). CD-HIT: accelerated for clustering the next-generation sequencing data. Bioinformatics.

[20] Langmead B, Trapnell C, Pop M, Salzberg SL (2009). Ultrafast and memory-efficient alignment of short DNA sequences to the human genome. Genome Biol.

[21] Li H, Handsaker B, Wysoker A, Fennell T, Ruan J (2009). The sequence alignment/map format and SAMtools. Bioinformatics.

[22] World Health Organization (2023). Statement on the update of WHO’s working deinitions and tracking system for SARS-CoV-2 variants of concern and variants of interest. https://www.who.int/news/item/16-03-2023-statement-on-the-update-of-who-s-working-definitions-and-tracking-system-for-sars-cov-2-variants-of-concern-and-variants-of-interest.

[23] Grubaugh ND, Gangavarapu K, Quick J, Matteson NL, De Jesus JG (2019). An amplicon-based sequencing framework for accurately measuring intrahost virus diversity using PrimalSeq and iVar. Genome Biol.

[24] R core team (2021). R: A Language and Environment for Statistical Computing. https://www.r-project.org/.

[25] Consorcio de Vigilancia Genómica de México (2023). CoViGen-Mex. http://mexcov2.ibt.unam.mx:8080/COVID-TRACKER/.

[26] Hadfield J, Megill C, Bell SM, Huddleston J, Potter B (2018). Nextstrain: real-time tracking of pathogen evolution. Bioinformatics.

[27] Aksamentov I, Roemer C, Hodcroft E, Neher R (2021). Nextclade: clade assignment, mutation calling and quality control for viral genomes. JOSS.

[28] Martin DP, Varsani A, Roumagnac P, Botha G, Maslamoney S (2021). RDP5: a computer program for analyzing recombination in, and removing signals of recombination from, nucleotide sequence datasets. Virus Evol.

[29] Schrörs B, Riesgo-Ferreiro P, Sorn P, Gudimella R, Bukur T (2021). Large-scale analysis of SARS-CoV-2 spike-glycoprotein mutants demonstrates the need for continuous screening of virus isolates. PLoS One.

[30] Taboada B, Zárate S, Iša P, Boukadida C, Vazquez-Perez JA (2021). Genetic analysis of SARS-CoV-2 variants in Mexico during the first year of the COVID-19 pandemic. Viruses.

[31] Zárate S, Taboada B, Muñoz-Medina JE, Iša P, Sanchez-Flores A (2022). The alpha variant (B.1.1.7) of SARS-CoV-2 failed to become dominant in Mexico. Microbiol Spectr.

[32] Taboada B, Zárate S, García-López R, Muñoz-Medina JE, Sanchez-Flores A (2022). Dominance of three sublineages of the SARS-CoV-2 Delta variant in Mexico. Viruses.

[33] Zárate S, Taboada B, Rosales-Rivera M, García-López R, Muñoz-Medina JE (2023). Omicron-BA.1 dispersion rates in Mexico varied according to the regional epidemic patterns and the diversity of local Delta subvariants. Viruses.

[34] McInerney P, Adams P, Hadi MZ (2014). Error rate comparison during polymerase chain reaction by DNA polymerase. Mol Biol Int.

[35] Pfeiffer F, Gröber C, Blank M, Händler K, Beyer M (2018). Systematic evaluation of error rates and causes in short samples in next-generation sequencing. Sci Rep.

[36] Markov PV, Ghafari M, Beer M, Lythgoe K, Simmonds P (2023). The evolution of SARS-CoV-2. Nat Rev Microbiol.

[37] Amicone M, Borges V, Alves MJ, Isidro J, Zé-Zé L (2022). Mutation rate of SARS-CoV-2 and emergence of mutators during experimental evolution. Evol Med Public Health.

[38] Tay JH, Porter AF, Wirth W, Duchene S (2022). The emergence of SARS-CoV-2 variants of concern is driven by acceleration of the substitution rate. Mol Biol Evol.

[39] García-López R, Rivera-Gutiérrez X, Rosales-Rivera M, Zárate S, Muñoz-Medina JE (2023). SARS-CoV-2 BW lineage, a fast-growing Omicron variant from southeast Mexico bearing relevant escape mutations. Infection.

