# Supplementary material for: Exploration of low-frequency allelic variants of SARS-CoV-2 genomes reveals coinfections in Mexico occurred during periods of VOCs turnover
Source: Microb Genom. 2024 Mar 21;10(3):001220. doi: 10.1099/mgen.0.001220 (PMC11004493; doi:10.1099/mgen.0.001220)
Supplement: Fig. S1. [file mgen-10-01220-s001.pdf]

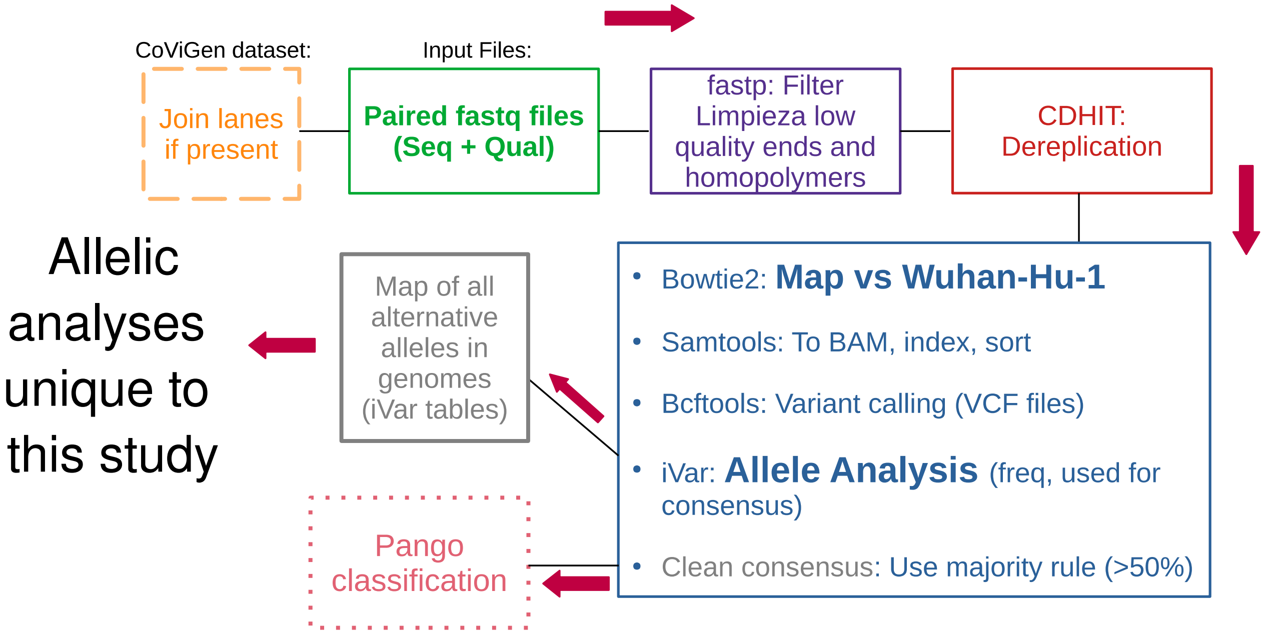

Figure S1. Bioinformatic workflow used at CoViGen-Mex institutions for genomic surveillance. The regular protocol ends with the Pango classification carried out with the consensus sequences. The branching path (gray box) is an alternative continuation for the detection of coinfections.

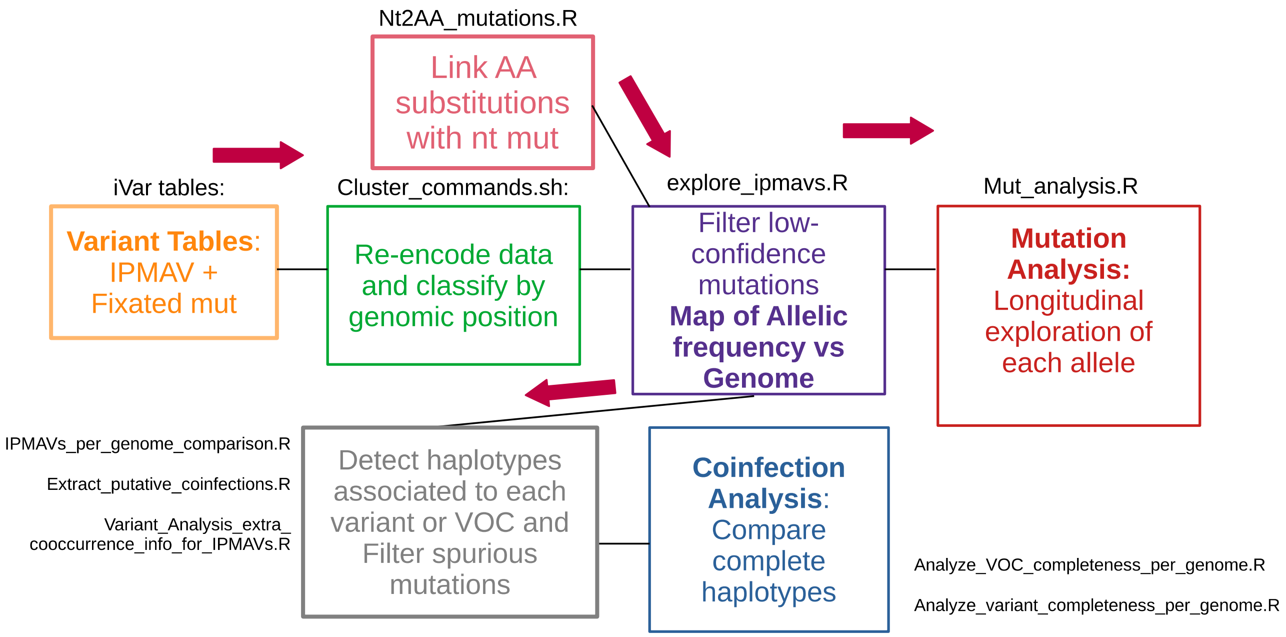

Figure S2. Bioinformatic workflow for mutation and coinfection analyses. This is a continuation of the standard CoViGen protocol for mapping allelic variants, comparing those seen as IPMAVs and calculating the completeness of each haplotype in coinfection analyses. The scripts shown in the diagram are available at https://github.com/rodrigogarlop/IPMAVs.

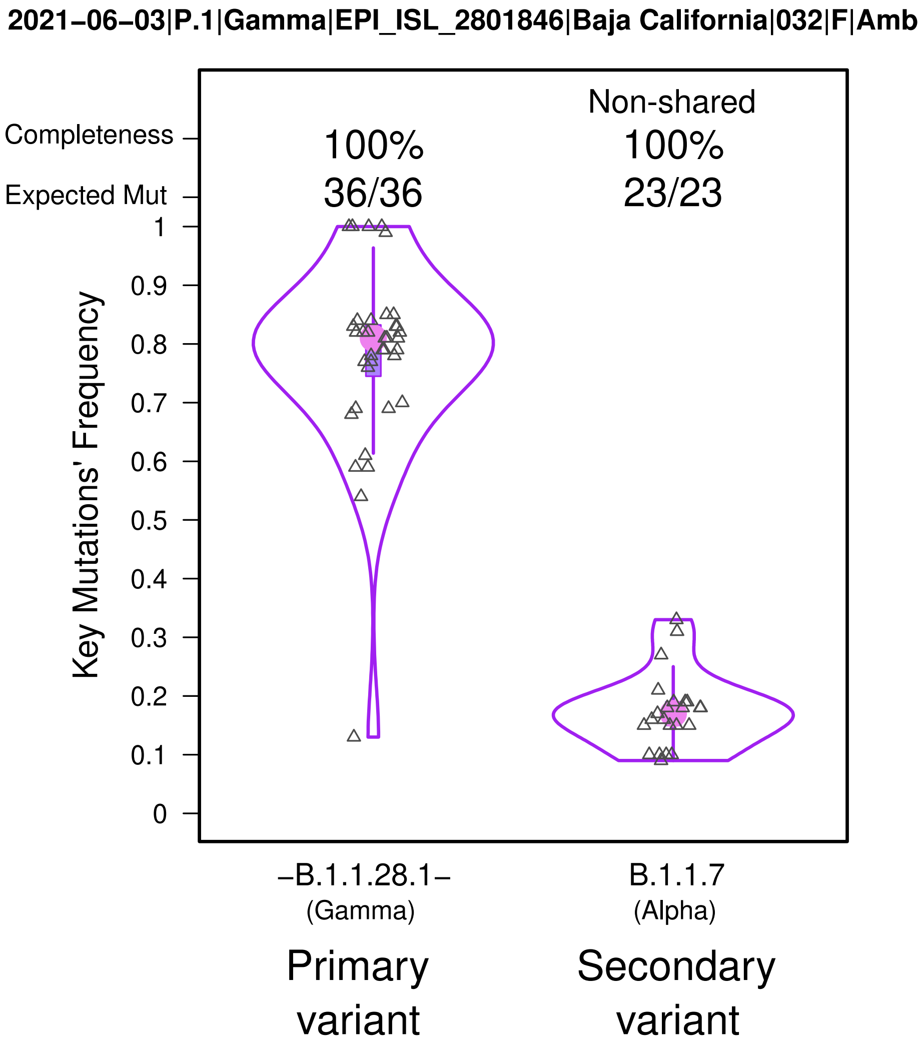

Figure S3. Example of a putative coinfection – Graphical representation. The example corresponds to a sample collected on June 3, 2021, from a female patient in Baja California. Violin plots show the overall frequency distribution of all mutations in a different haplotype, with each triangle showing the independent frequency of a different mutation. Pink bullets depict the distribution medians and the length of the violin shows the IQR. Completeness is shown on top, as well as the observed/expected mutation ratio. For the secondary variant, the ratio considers only mutations not theoretically shared with the primary variant, but the completeness is for the whole haplotype.
